# Magnetic-driven dynamic culture promotes osteogenesis of mesenchymal stem cell

**DOI:** 10.1186/s40643-021-00368-4

**Published:** 2021-02-13

**Authors:** Mengyang Hao, Minghao Xiong, Yangyang Liu, Wen-song Tan, Haibo Cai

**Affiliations:** State Key Laboratory of Bioreactor Engineering, East China University of Science and Technology, 130 Meilong Road, P. O. Box 309#, Shanghai, 200237 People’s Republic of China

**Keywords:** Dynamic culture, Osteogenesis, Magnetic fields, Mesenchymal stem cell, Scaffolds

## Abstract

Effective nutrient transport and appropriate mechanical stimulation play important roles in production of tissue-engineered bone grafts. In this study, an experimental set-up for magnetic-driven dynamic culture of cells was designed to mimic the microenvironment of the bone tissue. Here, its ability to contribute to osteogenic differentiation was investigated by inoculating human umbilical cord mesenchymal stem cells (HUMSCs) on magnetic scaffolds. The cytocompatibility of the developed magnetic scaffolds was verified for HUMSCs. Magnetic scaffolds seeded with HUMSCs were exposed to magnetic fields. The results showed that magnetic fields did not affect cell activity and promoted HUMSCs osteogenic differentiation. The magnetic scaffolds were magnetically driven for dynamic culture in the experimental set-up to evaluate the influence of HUMSCs osteoblast differentiation. The results indicated that magnetic-driven dynamic culture increased cell alkaline phosphatase (ALP) activity (*p* < 0.05) and calcium release (*p* < 0.05) compared with static culture. The effect was demonstrated in the expression of bone-associated genes. Overall, this study showed that magnetic-driven dynamic culture is a promising tool for regenerative bone engineering.

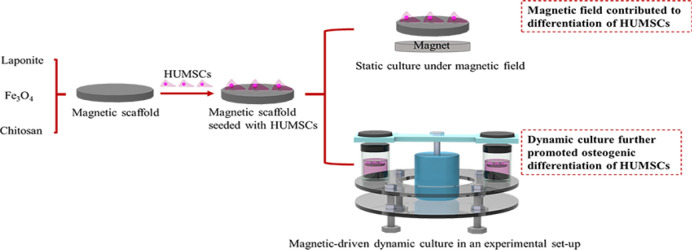

## Introduction

In the current rapid development of society, bone diseases caused by inflammation, tumors and accidents have become one of the major healthcare challenges worldwide (Li et al. 2018; Soundarya et al. 2018). Using human mesenchymal stem cells (MSCs) to repair damaged bone tissue is considered to be a reliable method (Toosi et al. 2018; Zhang et al. 2019). However, limited nutrients exchange in static bone tissue engineering culture might affect the differentiation of MSCs (Yeatts and Fisher 2011). A promising avenue to address these concerns may include the use of bioreactors to provide dynamic culture with different physical external stimuli (e.g., magnetic, electrical and mechanical stimulus) (Kumar et al. 2018; Mitra et al. 2017; Vetsch et al. 2015). Current tissue engineering bioreactors achieve dynamic culture by stirring or shaking (Beskardes et al. 2018; Wang et al. 2020b). However, this might produce additional problems, like the cell damage caused by excessive fluid shear stress (FSS), and the risk of damaging the scaffolds (Rodling et al. 2018). Hence, it is of great significance to realize dynamic cultivation by remote control.

Recently, magnetic fields have been incorporated in tissue engineering to promote the osteogenesis of MSCs by increasing the matrix vesicle secretion and mineralization (Chang et al. 2020). Some studies have shown that magnetic scaffolds driven by magnetic fields can realize dynamic culture (Filippi et al. 2019). However, the application of magnetic actuation in bone tissue engineering is rarely mentioned. Here, a magnetic-driven dynamic culture method using magnetic scaffolds was proposed, providing a novel way for dynamic culture without the damage to cells.

The combination of magnetic particles and hydrogels to prepare magnetic scaffolds has been extensively applied (Liu et al. 2016; Maharjan et al. 2021). Fe_3_O_4_ with high magnetic responsiveness is used to provide paramagnetism due to easy modification to scaffolds (Ge et al. 2019). Soft materials, like chitosan, are the base matrices for magnetic actuation in bone tissue engineering owing to their great encapsulation of magnetic particles (Ressler et al. 2018). In addition, the combination of chitosan and clay such as laponite improves the characteristics of scaffolds and cell adhesion (Gonzaga et al. 2020; Lee et al. 2019).

The purpose of this article was to evaluate the effect of magnetic-driven dynamic culture on the osteogenesis of HUMSCs via an experimental set-up. First, a series of magnetic scaffolds with great biocompatibility and high magnetic responsiveness were synthesized. Second, the HUMSCs were inoculated on magnetic scaffolds and the effects of magnetic fields on the proliferation and osteogenic differentiation of HUMSCs were studied. Finally, the HUMSCs were seeded on magnetic scaffolds and cultured in the experimental set-up to investigate its effect on HUMSCs proliferation and osteogenic differentiation.

## Materials and methods

### Magnetic field exposure system

**Fig. 1:**
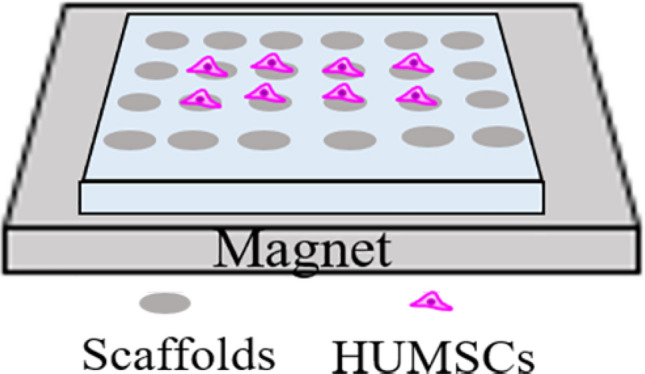
Schematic representation of magnetic field exposure system

As shown in Fig. 1, the magnetic field exposure system was built with a neodymium cuboid magnet (150 mm long × 100 mm wide × 25 mm thick; Dongguan Yanghua Magnets, Guangdong, China). The cells were exposed to magnetic fields of different intensities by changing the position of the scaffolds. In this paper, three intensities were investigated: 5, 20 and 50 mT. A Gauss meter (TM-701, Kanetec, Tokyo, Japan) was used to measure the magnetic field intensity.

### Experimental set-up

**Fig. 2:**
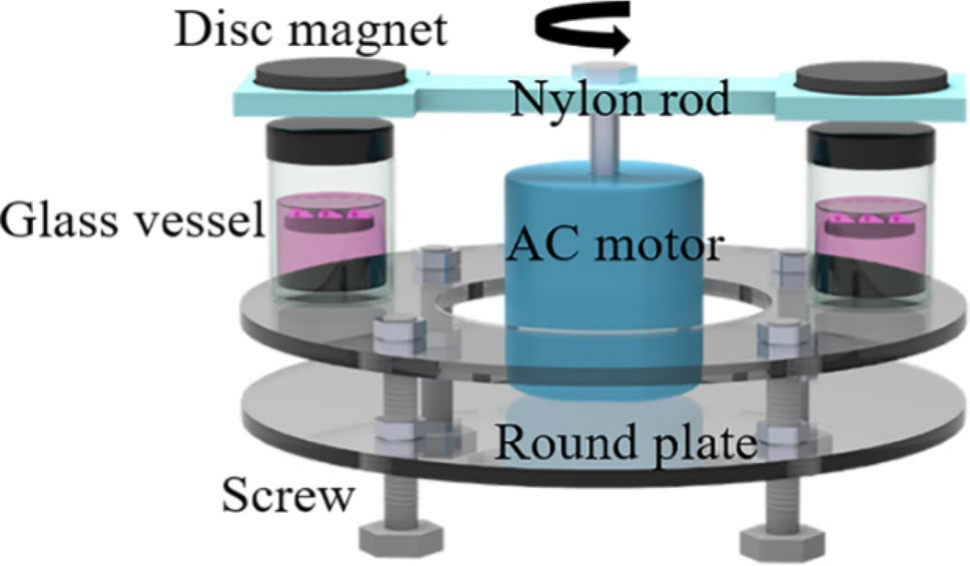
Schematic representation of the experimental set-up

As shown in Fig. 2, the supporting platform for the experimental set-up was made of two polymethyl methacrylate (PMMA) plates. Two disc magnets (50 mm diameter × 10 mm thick; Yanghua Magnets, Guangdong, China) were placed on a nylon rod to generate dynamic magnetic fields. The AC motor (Shenzhen Xinda Motors, Guangdong, China) drove the nylon rod with two disc magnets at both sides to rotate above the glass vessels. The stainless steel threaded rods were used for magnetic field intensity adjustment. Motor speed: 5 rpm.

### Preparation of scaffolds

The scaffolds were prepared by a freeze-drying method. In brief, 2 g chitosan (Sigma-Aldrich, Louis, MO) was dissolved in 100 ml 1.0% (v/v) acetic acid. The laponite (Laporte Industries, Luton, UK) and Fe_3_O_4_ (Sigma-Aldrich, Louis, MO) with different weight ratios were added to the solution as reported in Table 1. The solutions were frozen in 24-well Petri dishes for 24 h and lyophilized to remove water for 48 h. The scaffolds were immersed with 0.1 M NaOH to remove excess acetic acid for 4 h, and washed three times with distilled water. Finally, the scaffolds were lyophilized again.

**Table 1:** The formula of scaffolds

Scaffold ID	S0	S1	S2	S3	S4
Fe_3_O_4_% (w/w)	0	0.4	0.6	0.4	0.6
Laponite % (w/w)	0	0.4	0.4	0.6	0.6

### Characterization of magnetic responsiveness

**Fig. 3:**
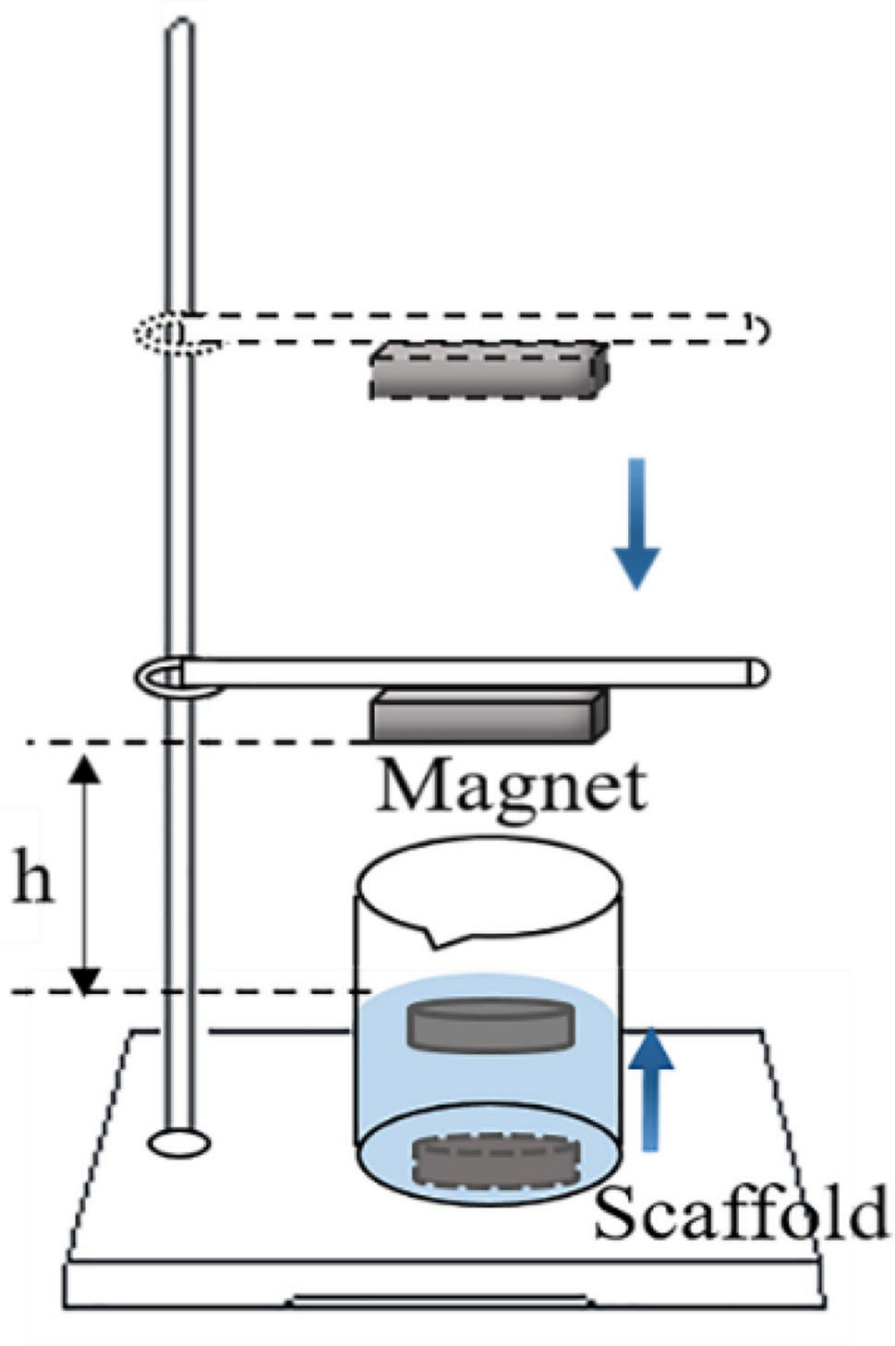
Magnetic responsiveness of the scaffolds measurement. h is the distance between the magnet and the liquid level when the scaffolds approached the liquid level

The magnetic responsiveness of the scaffolds was characterized by distance. As shown in Fig. 3, the distance was measured using the following procedure. The S0–S4 scaffolds of the same size were immersed in distilled water for 24 h. By magnetic attraction of a gradual descending magnet, the submerged scaffolds surfaced. The distance (h) between the magnet and the liquid level was measured. The magnetic scaffolds were subjected to gravity, buoyancy and magnetic force in distilled water. The magnetic responsiveness of the scaffolds was positively correlated with the distance (h) when the required magnetic force was determined. In this paper, both buoyancy and gravity of different scaffolds were approximately equal due to the weight difference of scaffolds of the same size that could be disregarded. A scale was used to measure the distance.

### Fourier-transformed infrared spectroscopy (FTIR)

A continuous scanning FTIR spectrometer (Jasco-4100, JASCO, Japan) was used to observe the chemical status of the samples. FTIR spectrum analysis was conducted in the wavelength range of 400–4000 cm^−1^ using a potassium bromide pellet technique.

### X-ray diffraction (XRD)

The diffraction analysis of the samples was carried out by XRD (Ultima IV, Rigaku, Japan) at a voltage of 40 kV. The XRD patterns were recorded in the range 2θ = 5 − 90°

### Scanning electron microscopy (SEM)

The microstructure of cells and scaffolds was observed by SEM (Hitachi S-3400N; Hitachi Ltd, Tokyo, Japan) at 3 kV. To observe the cell morphology on the surface of scaffolds, the samples were treated with 2.5% (w/v) glutaraldehyde solution for 24 h. Then, dehydration of the samples was conducted through different volume fraction ethanol (10, 20, 40, 50, 70, 90, and 100% ethanol) for 30 min. Before observation, the samples were treated with gold sputtering for 50 s.

### Porosity measurement

Porosity was measured by changes in liquid volume. Briefly, the scaffolds were immersed in a known volume (*V*_1_) ethanol graduated cylinder until no bubbles were generated. The total volume of the soaked scaffolds and ethanol was recorded as *V*_2_, and the volume of residual alcohol after the scaffolds were removed was recorded as *V*_3_. Porosity was calculated using the formula:

$${\text{Porosity}} = \left( {V_1 - V_3 } \right)/\left( {V_2 - V_3 } \right) \times 100 \, \% .$$

### Swelling measurement

The following procedure was used to evaluate the swelling ratio and water retention ratio of the scaffolds. The freeze-dried scaffolds were weighed (*W*_1_) and immersed in distilled water for 1 day, then the wet scaffolds were taken out and the surface water was sucked dry and weighed (*W*_2_). After that, the wet scaffolds were centrifuged (500 rpm, 3 min) and weighed again (*W*_3_). The swelling and water retention ratio of scaffolds were calculated using the formulas:

$${\text{Swelling}}\,{\text{ratio}} = \left( {W_2 - \, W_1 } \right)/W_1 ,$$

$${\text{Water}}\,{\text{retention}}\,{\text{ratio}} = \left( {W_3 - \, W_1 } \right)/W_1 .$$

### Proliferation of HUMSCs on scaffolds

Before cell seeding, both sides of the scaffolds were sterilized under ultraviolet radiation for 12 h. 2 × 10^4^ HUMSCs were inoculated on each scaffold and cultured for 3 days. After, the cytotoxicity of the scaffolds to HUMSCs was determined using a MTT assay. The F-actin in HUMSCs stained with Rhodamine-Phalloidin was observed using a confocal laser scanning microscope (CLSM; TCS SP8, Leica). 1 × 10^4^ HUMSCs were seeded on each scaffold for 1, 5, 7 days. HUMSCs proliferation was determined using a CCK-8 assay. CLSM was utilized to observe live/dead cells stained with calcein-AM and PI.

### Osteogenic differentiation of HUMSCs on scaffolds

2 × 10^4^ HUMSCs were inoculated on each scaffold and cultured in the presence of osteogenic induction factors. After 7 days of osteogenic induction, the ALP activity was measured by a BCIP/NBT alkaline phosphatase color development kit (Beyotime, China). To further determine mineralization, the calcium analysis was carried out by a calcium quantitative kit (Nanjing Jiancheng, China) after 14 days of osteogenic induction.

### Osteogenesis-related genes expression analysis

2 × 10^5^ HUMSCs were inoculated on each scaffold and cultured in the presence of osteogenic induction factors. After 14 days of osteogenic induction, total RNA was extracted after treatment with TRIzol reagent (Invitrogen, Carlsbad, CA, USA). The cDNA converted from Total RNA was carried out by a kit (iScript cDNA synthesis) from BioRad (Bio-Rad, Hercules, CA, USA). Reverse transcriptase real-time (quantitative) polymerase chain reaction (RT-qPCR) was carried out by a Quant Studio 3 real-time PCR system (Applied Biosystems, Foster City, CA, USA) and SYBR reagent (Takara Bio Inc., Kyoto, Japan). The primer sequences are presented in Table 2. The gene expression was calculated using the 2^−ΔΔCt^ method.

**Table 2:** RT-PCR primer sequences

Gene	Forward Primer 5′ → 3′	Reverse Primer 5′ → 3′
GAPDH	GAAGGTGAAGGTCGGAGT	GAAGATGGTGATGGGATTT
ALP	CTCCTCGGAAGACACTCT	AGACTGCGCCTGGTAGTTG
OCN	GCGGTGCAGAGTCCAGCAAA	CCCTAGACCGGGCCGTAGAA
Col-I	CCTCTGCGGGACTCAACAAC	AGCCCATTAGTGCTTGTAAAGG

### Statistical analysis

Statistical significance of all data was carried out using SPSS 20.0 software. The data were expressed as mean ± standard deviation. A one-way ANOVA was used for multiple group comparisons. The significance level was set at *p* < 0.05.

## Results

### Characterization of magnetic scaffolds

**Fig. 4:**
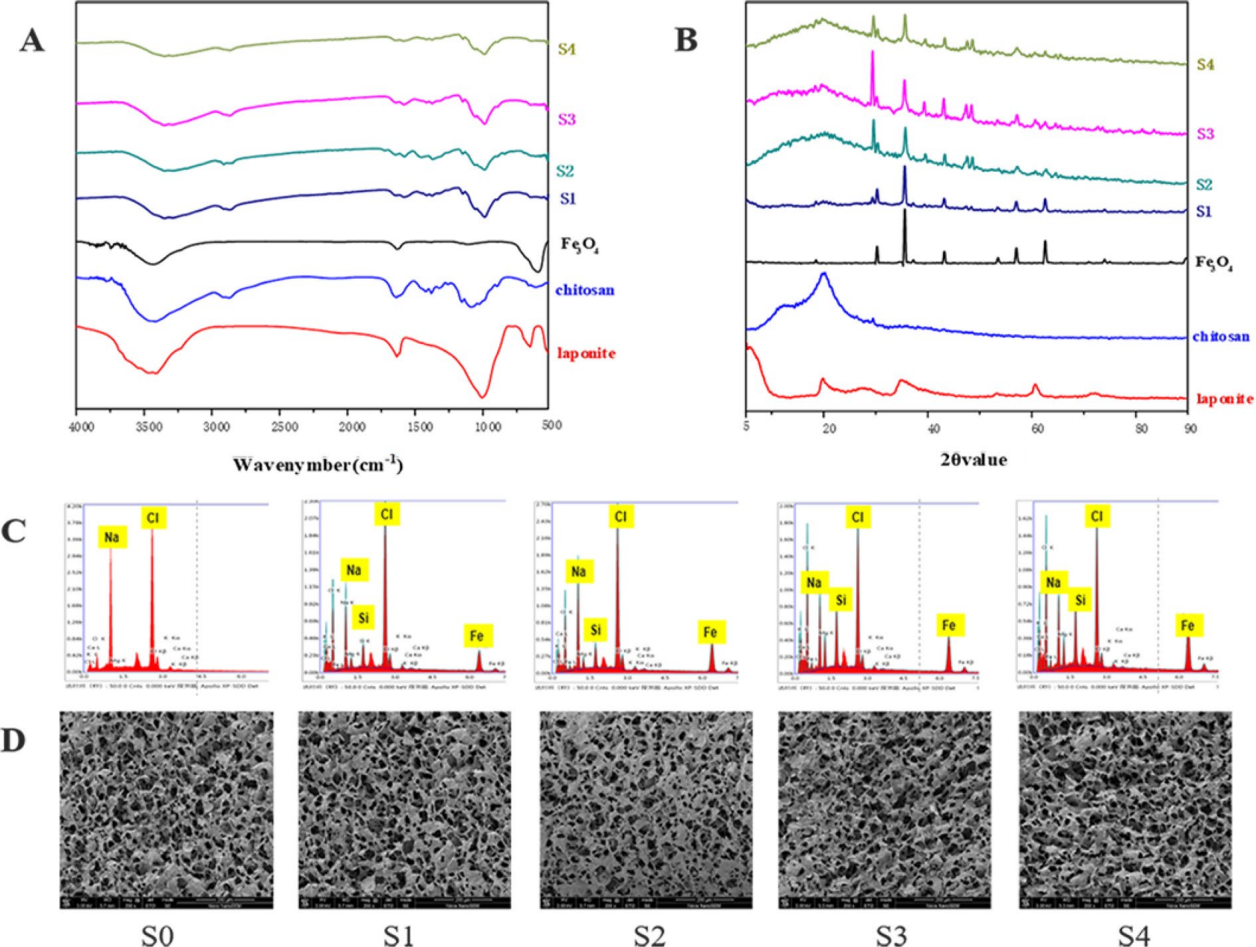
Characterization of scaffolds. **A** FTIR spectra of chitosan, laponite, Fe_3_O_4_ and scaffolds. **B** XRD spectra of chitosan, laponite, Fe_3_O_4_ and scaffolds. **C** EDS of scaffolds. **D** SEM images of scaffolds. Scale bar: 200 μm

Magnetic scaffolds with great biocompatibility and sensitive magnetic responsiveness were prepared by freeze-drying technique. FTIR was carried out to observe the chemical status of the scaffolds to determine the composition of scaffolds. As shown in Fig. 4A, the FTIR spectrum revealed characteristic peaks of S1–S4 scaffolds at 590 cm^−1^ (Fe–O, stretching), 1000 cm^−1^ (Si–O-Si, stretching), 1640 cm^−1^ (amide II band N–H, stretching), 2880 cm^−1^ (C–H, stretching), 3360 cm^−1^ (O–H and N–H, stretching). The FTIR spectra of magnetic scaffolds had the characteristic peaks of chitosan, Fe_3_O_4_, and laponite.

Crystallinity contributes to the mechanical and tensile strength of the scaffolds. The XRD was used to measure the crystallinity of scaffolds. The XRD spectrum of the S1-S4 scaffolds demonstrated the characteristic diffraction peaks at 20.6°, 30.2°, 35.5°, 43.2°, 53.6°, 57.0°, which indicated the semi-crystalline nature of chitosan and Fe_3_O_4_ (Fig. 4B). The strong diffraction peaks of the laponite at 19.42°, 34.99°, 61.03°, 72.58°, indicate the crystalline nature of laponite. However, no such peaks were seen for S1–S4 scaffolds, implying that laponite was amorphized during the preparation. The EDS analysis further revealed the elemental component peaks of scaffolds. Fe and Si elements were detected on the surface of the S1–S4 scaffolds (Fig. 4C).

Uniform porous structure is necessary for cells to adhere and proliferate on scaffolds. The SEM was used to observe the microstructure of scaffolds. There was no significant difference in structure and morphology between S0 and S1–S4 scaffolds (Fig. 4D). This indicated that the introduction of laponite and Fe_3_O_4_ did not affect the microstructure of the scaffolds.

**Fig. 5:**
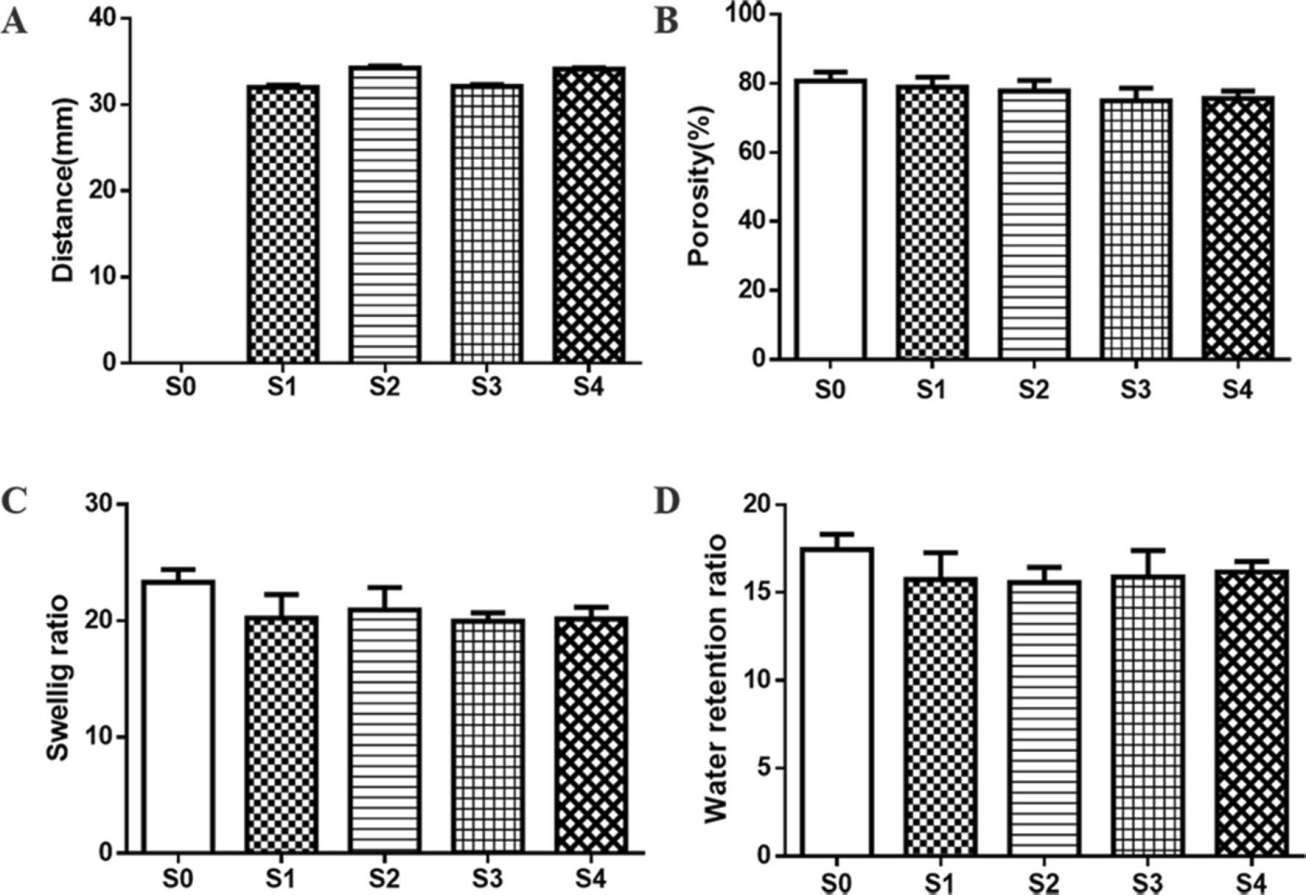
Analysis of physicochemical properties of scaffolds. **A** The distance between the magnet and the liquid level. The magnetic responsiveness of the scaffolds was positively correlated with the distance. **B** The porosity of scaffolds. **C** The swelling ratio of scaffolds. **D** The water retention ratio of scaffolds

High magnetic responsiveness is the basic condition of magnetic-driven dynamic culture. The magnetic responsiveness of the scaffolds was characterized by the distance (h) between the magnet and the liquid level. When the S1–S4 scaffolds were close to the liquid level, the distances (h) of S1 and S3 scaffolds were about 32.1 mm, and that of S2 and S4 scaffolds were about 34.2 mm (Fig. 5A). These results demonstrated that scaffolds with higher Fe_3_O_4_ content had stronger magnetic responsiveness.

The physicochemical properties of scaffolds have a great effect on their ability to culture cells. The similarity of porosity, swelling ratio and water retention ratio of scaffolds are shown in Fig. 5B–D, respectively. These results suggested that the physicochemical properties of the scaffolds did not change after adding Fe_3_O_4_ and laponite.

### Cells proliferation on magnetic scaffolds

**Fig. 6:**
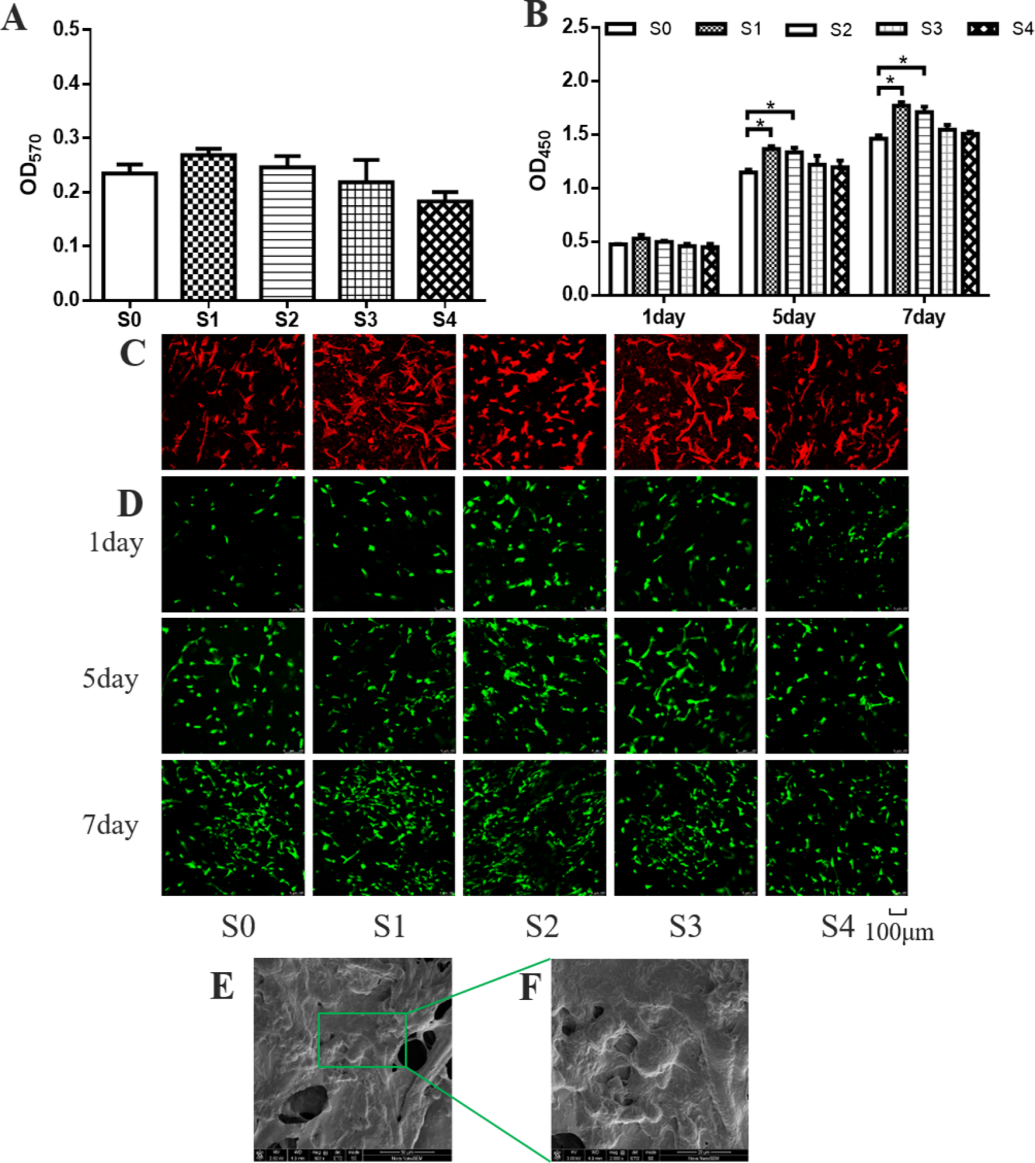
Cells proliferation on scaffolds. **A** The HUMSCs viability on the scaffolds after 3 days. **B** The HUMSCs proliferation after seeding on the scaffolds for 1, 5, and 7 days. **C** Skeleton of the HUMSCs on the scaffolds after 3 days. **D** The live/dead staining of HUMSCs after seeding on the scaffolds for 1, 5, and 7 days. **E** SEM images of the HUMSCs after seeding on the S1 scaffolds for 7 days. Scale bar: 50 μm. **F** were that of the area framed by green. Scale bar: 20 μm

To evaluate the cytocompatibility of magnetic scaffolds for HUMSCs, HUMSCs were cultured on scaffolds with or without laponite and Fe_3_O_4_. After 3 days of culture, the MTT assay was used to test the toxicity of scaffolds. The cell activity of S0 and S1–S4 scaffolds was similar. This indicated that magnetic scaffolds were less likely to cause toxicity to HUMSCs (Fig. 6A). In addition, the well-defined actin fibers were observed on the S0 and S1–S4 scaffolds (Fig. 6C). These results indicated magnetic scaffolds had no cytotoxicity.

The proliferation of HUMSCs was detected after cultured on S0–S4 scaffolds for 1, 5 and 7 days. As shown in Fig. 6B, the results showed that the proliferation rates of HUMSCs on S0 and S1–S4 scaffolds were similar on day 1. However, after 5 days of incubation, the proliferation rates of S1–S2 scaffolds were higher than that of S0 scaffolds. The proliferation rates of S3–S4 scaffolds were not improved compared with S0 scaffolds. These results revealed that the introduction of laponite and Fe_3_O_4_ promoted cell proliferation, but this effect would be weakened with the increase of laponite content.

The viability of HUMSCs cultured on S0–S4 scaffolds for 1, 5, and 7 days was determined by live/dead staining. The number of viable HUMSCs on the S0–S4 scaffolds increased gradually over time, indicating that the S0–S4 scaffolds could maintain the vitality of HUMSCs (Fig. 6D). These results largely followed the same trends as cell proliferation (Fig. 6B). To further explore the morphology of HUMSCs on the scaffolds, we scanned cells on the S1 scaffold surface using SEM. HUMSCs were homogeneously distributed on the S1 scaffolds. Besides, HUMSCs developed cytoplasmic extensions, attaching to the porous surface (Fig. 6E). These results suggested that the magnetic scaffolds had a capacity for maintaining cell proliferation.

### Effect of magnetic fields on HUMSCs proliferation and osteogenic differentiation

**Fig. 7:**
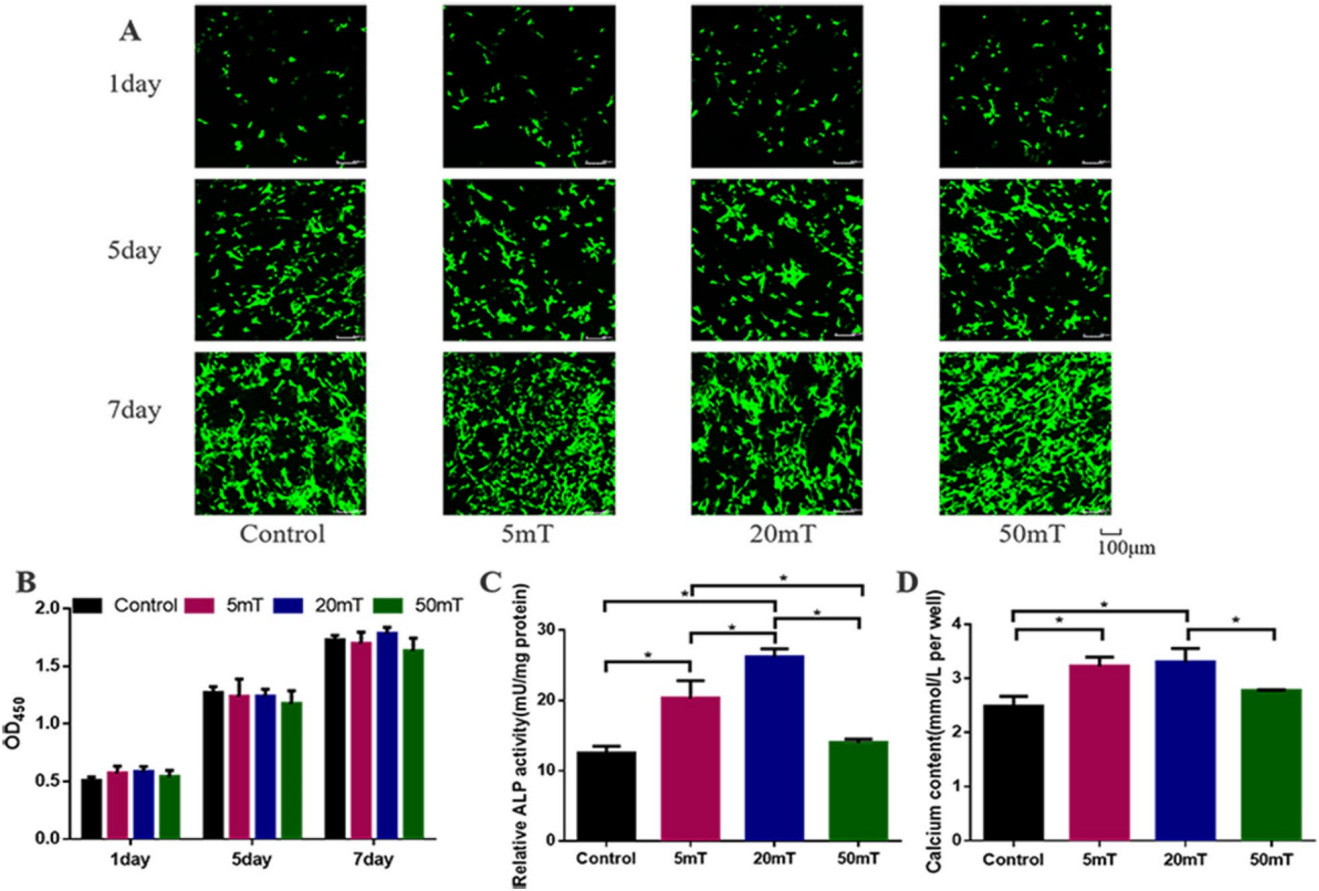
Effect of magnetic fields on HUMSCs proliferation and osteogenic differentiation **A** The live/dead staining of HUMSCs after seeding on the S1 scaffolds for 1, 5, and 7 days. **B** The HUMSCs proliferation after seeding on the S1 scaffolds for 1, 5, and 7 days. **C** Quantitative analysis of ALP activity in HUMSCs cultured for 7 days. **D** Calcium contents in HUMSCs cultured for 14 days

To study the effect of magnetic fields on cell proliferation, HUMSCs were cultured on the S1 scaffolds under 5, 20, and 50 mT magnetic fields, respectively. No magnetic field was used as a control group. No effect of the magnetic fields on HUMSCs proliferation was seen after 1, 5 and 7 days of culture (Fig. 7B). The effect of magnetic fields on HUMSCs vitality was evaluated by live/dead staining. The results showed that living cells increased with time, indicating that magnetic fields did not affect cell activity (Fig. 7A).

To evaluate the effect of magnetic fields on osteogenic differentiation of HUMSCs, cell ALP expression and calcium content were detected at the cellular level. As shown in Fig. 7C, D, HUMSCs under 5 and 20 mT magnetic fields showed higher ALP expression compared with the control group. In addition, ALP expression was strongest under 20 mT magnetic field. The content of calcium under 5 and 20 mT magnetic fields was significantly increased. However, ALP expression and calcium content under 50 mT magnetic field were similar to that in the control group (Fig. 7C). Collectively, these results demonstrated that magnetic fields had no effect on the proliferation of cells seeded on the magnetic scaffolds and promoted osteogenic differentiation of HUMSCs.

### Effect of dynamic culture on HUMSCs proliferation and differentiation

**Fig. 8:**
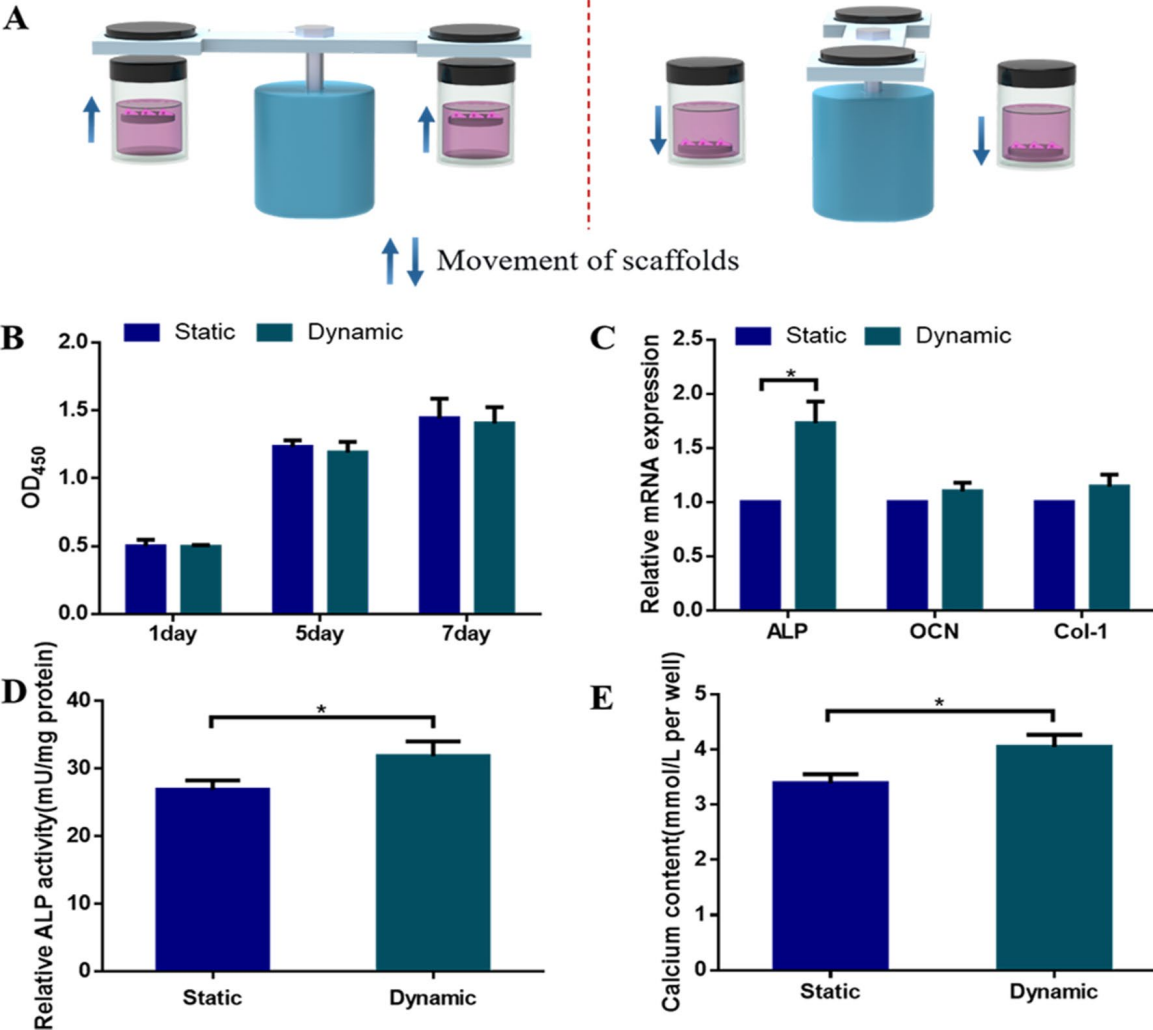
Proliferation and differentiation of HMUSCs in dynamic and static cultures. **A** The working principle of the experimental set-up. The magnetic scaffolds move up and down under the action of magnetic force and gravity. **B** The HUMSCs proliferation after seeding on the S1 scaffolds for 1, 5, and 7 days. **C** The quantitative evaluation of osteogenic-related genes in HUMSCs cultured for 14 days. **D** Quantitative analysis of ALP activity in HUMSCs cultured for 7 days. **E** Calcium contents in HUMSCs cultured for 14 days

To evaluate the effects of dynamic culture on HUMSCs proliferation and differentiation, the S1 scaffolds seeded with HUMSCs were dynamically cultured in the experimental set-up. Static culture under 20 mT magnetic field was used as a control group. The motion was obtained by changing the position of the magnetic scaffold. The disc magnets attracted the magnetic scaffolds to the liquid level. When the disc magnets were turned away, the magnetic scaffolds that lost magnetic attraction returned to the bottom of the vessels. (Fig. 8A). In this study, we chose 20 mT as the magnetic field intensity at the bottom of the vessels when the magnet was directly above.

After 1, 5, and 7 days of culture, no significant difference in cell proliferation between static and dynamic scaffolds was observed (Fig. 8B). The effects of dynamic culture on osteogenic differentiation were evaluated at the molecular and cellular levels. At the molecular level, the expression of osteogenic genes including ALP, osteocalcin (OCN) and type I collagen (Col-I) was assayed after 14 days of culture. The mRNA expression level of ALP in dynamic culture was significantly higher than that in static culture. The mRNA expression levels of OCN and Col-I in the dynamic culture were slightly higher than that in the static group, but there was no significant difference (Fig. 8C).

At the cellular level, dynamic culture significantly improved ALP expression compared with that of the static group after 7 days of culture (Fig. 8D). Moreover, compared with static-cultured scaffolds, the calcium phosphate deposition was significantly increased when HUMSCs were cultured on dynamic-cultured scaffolds after 14 days of culture (Fig. 8E). These results demonstrated that dynamic culture further promoted osteogenic differentiation of HUMSCs.

## Discussion

Current bone tissue engineering strategies to promote bone regeneration include modification of scaffolding materials and applying external stimulation (Kang et al. 2013; Li et al. 2020a; Wang et al. 2020a). In this study, we designed an experimental set-up based on magnetic actuation to stimulate the osteogenic differentiation of MSCs while reducing cell damage by remote control. The magnetic stimulation and FSS provided by the up–down movement of the magnetic scaffolds might be factors influencing the osteogenic differentiation of MSCs.

The effect of magnetic fields on the osteogenic differentiation of MSCs is considered to be associated with type, intensity, frequency, and duration (Li et al. 2020b; Liu et al. 2013; Yu et al. 2014). Magnetic fields include static magnetic fields and dynamic magnetic fields. The likely mechanisms of static magnetic fields regulating cell function include: first, Hall voltage generated by magnetic fields affects charged ions and changes streaming potential; second, magnetic fields with sufficiently large gradient act on diamagnetic materials to generate magneto-mechanical force; third, magnetic fields affect chemical reactions in cells by affecting the radical pair of reaction intermediates (Xia et al. 2018). Dynamic magnetic fields, including pulsed electromagnetic fields, sinusoidal electromagnetic fields, alternating electromagnetic fields and rotating magnetic fields, have been proved to promote bone formation. Some studies have shown that pulsed magnetic fields increased the cytosolic Ca^2+^ and activation of calmodulin (Wang et al. 2016). However, the exact mechanisms of other types of dynamic magnetic fields in bone formation remain unclear. Different parameters correspond to different magnetic fields, and cells have different responses to different magnetic fields, including the change of intracellular iron content (Wang et al. 2016). In this study, the magnetic nanoparticle Fe_3_O_4_ was introduced to provide magnetic response for the scaffolds and realize the movement in the device. The effect of dynamic magnetic fields provided in this study on the osteogenesis of MSCs will be investigated in further research.

In this study, the strength and loading time of FSS were determined by magnetic field strength and the rotating speed of the permanent magnet. It has been considered that FSS promotes the osteogenic differentiation of MSCs in three steps: cells detect physical stimulation through ion channels and integrins; mechanical signals are transformed into intracellular biochemical signals through matrix metalloproteinases; biochemical signals activate transcription factors to regulate the expression of osteogenic genes, such as ALP, OCN, COL-I (Liu et al. 2010). MSCs differentiate into osteoblasts as a result of the expression of osteogenic-related genes. To explore the best experimental conditions, the effects of FSS strength and loading time on osteogenic differentiation will be investigated in further research.

This paper demonstrated that magnetic-driven dynamic culture can promote osteogenesis of MSCs, and that magnetic-driven dynamic culture provided both mechanical and magnetic stimulation for cells to mimic the micro and macro‐environments of the bone tissues. Overall, the magnetic attraction will be a new idea to design tissue engineering bioreactor and the experimental set-up can be used as a prototype for the future design of bone tissue engineering bioreactor.

## Conclusion

Through introducing laponite and Fe_3_O_4_ into chitosan, magnetic scaffolds that promoted the osteoblast differentiation of HUMSCs under external magnetic fields were prepared. The use of this experimental set-up enhanced the osteogenic differentiation of HUMSCs, which will have important guiding significance for the design of bone tissue engineering bioreactor.

## Data Availability

All data generated or analyzed during this study are included in this published article.

## Abbreviations

HUMSCs: Human umbilical cord mesenchymal stem cells
ALP: Alkaline phosphatase
MSCs: Mesenchymal stem cells
FSS: Fluid shear stress
PMMA: Poly (methyl methacrylate)
FTIR: Fourier-transformed infrared spectroscopy
XRD: X-ray diffraction
SEM: Scanning electron microscopy
CLSM: Confocal laser scanning microscope
RT-qPCR: Reverse transcriptase real-time (quantitative) polymerase chain reaction
OCN: Osteocalcin
Col-I: Type I collagen
